# Medical Management of Recurrent Left Ventricular Assist Device Thrombosis in a Patient With Biventricular Assist Devices

**DOI:** 10.7759/cureus.11872

**Published:** 2020-12-03

**Authors:** Laura Onderko, Sean H Novak, Sanjeev A Francis, Esther S Shao, Maxwell Afari

**Affiliations:** 1 Cardiology, Maine Medical Center, Portland, USA; 2 Radiology, Maine Medical Center, Portland, USA

**Keywords:** ventricular assist device, thrombosis, thrombolytic

## Abstract

Ventricular assist device (VAD) pump thrombosis is a known complication and while the preferred standard treatment is surgical pump exchange this procedure is not without risk and for some patients the risks are prohibitive. This is a case of a 68-year-old female with bilateral HeartWare ventricular assist devices (HVAD) implanted as destination therapy who presented with signs of recurrent pump thrombosis. Surgical pump exchange was deemed to confer prohibitive risk due to her underlying medical co-morbidities and therefore not an option for treatment. After careful consideration of possible options for treatment, she received systemic thrombolysis (Alteplase 5 mg IV bolus followed by 3 mg/hour infusion for 10 hours through a central line) which was successful. This case highlights, not only the rarity of bilateral VADs as destination therapy, but also demonstrates the safety and efficacy of using systemic thrombolytics in patients with bilateral HVADs for treatment of pump thrombosis.

## Introduction

In advanced heart failure patients, heart transplantation is considered the “gold standard” therapy but this option is limited by the availability of donor hearts. Mechanical circulatory support devices, such as left ventricular assist devices (LVAD) serve as a bridge-to-transplant or as destination therapy for those who do not qualify for or have limited access to donor hearts. The use of HeartWare ventricular assist device (HVAD) as a right ventricular assist device (RVAD) is considered for off-label use, and biventricular assist devices (BIVAD) are currently not FDA approved for ambulatory use. On review of current literature, case reviews of long-term BIVAD use are reported as a bridge to transplant, not as a destination therapy [1]. In addition, the Seventh Interagency Registry for Mechanically Assisted Circulatory Support (INTERMACS) Report with data from 2012 to 2014 showed a one-year survival for BIVAD of 56% [2]. Management of thromboembolism, bleeding events, and other complications associated with LVADs can be challenging. Pump exchange is the definitive treatment for pump thrombosis; however, it is not benign. The Sixth Annual INTERMACS report showed that one-year survival after the initial implant is close to 80%, however, falls to 65% after the second implant [3]. A more conservative approach is often pursued with medical treatment instead with options including catheter-directed lysis, systemic anticoagulation, or systemic thrombolysis [4,5]. We present a rare case of an ambulatory biventricular HVAD patient who presented twice with suspected pump thrombosis and was treated with systemic thrombolytics.

## Case presentation

History of presentation

A 68-year-old female with bilateral HVADs presented with elevated lactate dehydrogenase (LDH) >2.5 times the upper limit of normal with corresponding power spikes, concerning pump thrombosis. She was admitted to the cardiac intensive care unit for workup and management.

Past medical history

She had a prior history of severe cardiogenic shock in the setting of ST-elevation myocardial infarction (STEMI) requiring Veno-arterial extracorporeal membrane oxygenation (VA-ECMO) which was later transitioned to an LVAD. LVAD implantation was complicated by right ventricular failure for which she received an HVAD implanted as an RVAD [LO1]. She was listed for transplant; however, her post-op course was complicated by spinal cord infarction causing paraplegia leading to her removal from the transplant list. Due to the history of recurrent severe gastrointestinal bleeding, her international normalized ratio (INR) goal was adjusted to 1.8-2.5 and she was started on thalidomide.

Investigations

Admission 1

**Figure 1:**
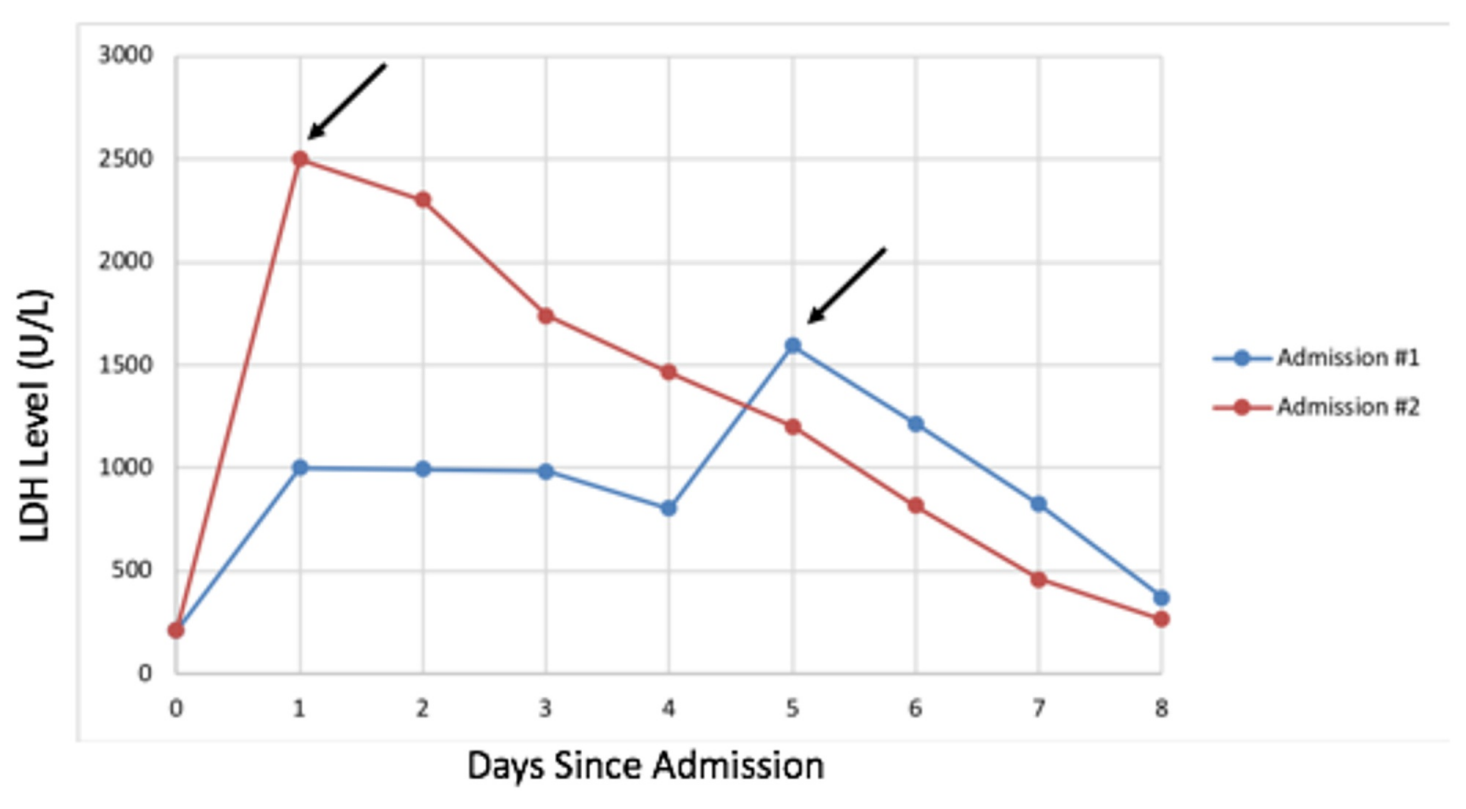
Demonstrates the LDH trend from the first admission compared to her second admission for pump thrombosis. Arrows indicates when lytics were administered for both admission #1 and admission #2. Lytics were given on day 7 for first admission (LDH on that day was 1594 U/L) and day 1 for second admission (LDH on that day was >2500 U/L). LDH: lactate dehydrogenase.

She presented for a routine clinic visit and lab work was significant for an LDH of 1000 U/L. She was otherwise asymptomatic and hemodynamically stable with mean arterial pressure (MAP) in the 80s. The patient’s baseline LDH trend was 180-200 U/L. INR was 3.0. As shown in Figure 1, LDH initially downtrended with intensive heparin therapy until day 7, when there was an upswing of LDH to 1594 U/L. Plasma-free hemoglobin was also found to be elevated at 166 mg/dL (normal 0-4.9 mg/dL). The patient also developed new hematuria. This correlated with LVAD power spikes of approximately 5 W with low flow. The RVAD was also interrogated and showed no alarms or increased power. Following administration of alteplase (5 mg IV bolus of alteplase followed by a 3 mg/hour infusion for 10 hours given through a central line), LDH began to downtrend and by the time of discharge was 372 U/L (Figure 1).

Admission 2

**Figure 2:**
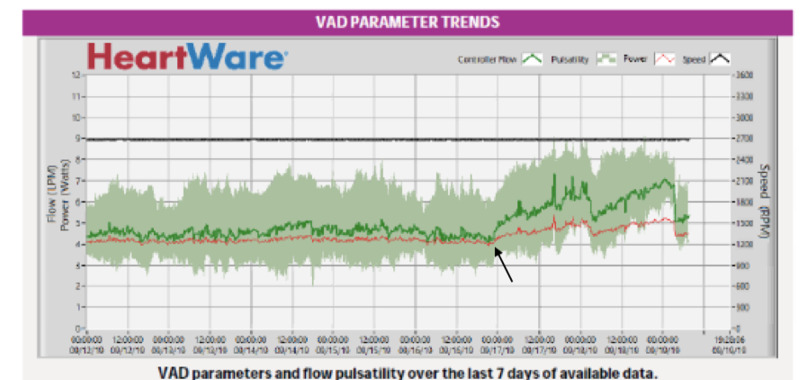
Demonstrates LVAD power trend leading up to her second admission for pump thrombosis. Arrow indicates increase in power trend suggestive of pump thrombosis. LVAD: left ventricular assist device.

She presented a month later with signs of recurrent pump thrombosis of the LVAD with LDH up to 2500 U/L and LVAD power spikes > 7 W (Figure 2). She was again overall hemodynamically stable, but with symptoms of increased fatigue and hematuria. INR on admission was 2.2. She again received alteplase in the same bolus and then infusion doses as in admission 1 via a central line, which resulted in LDH trending down to less than 500 U/L by the time of discharge as shown in Figure 1.

**Figure 3:**
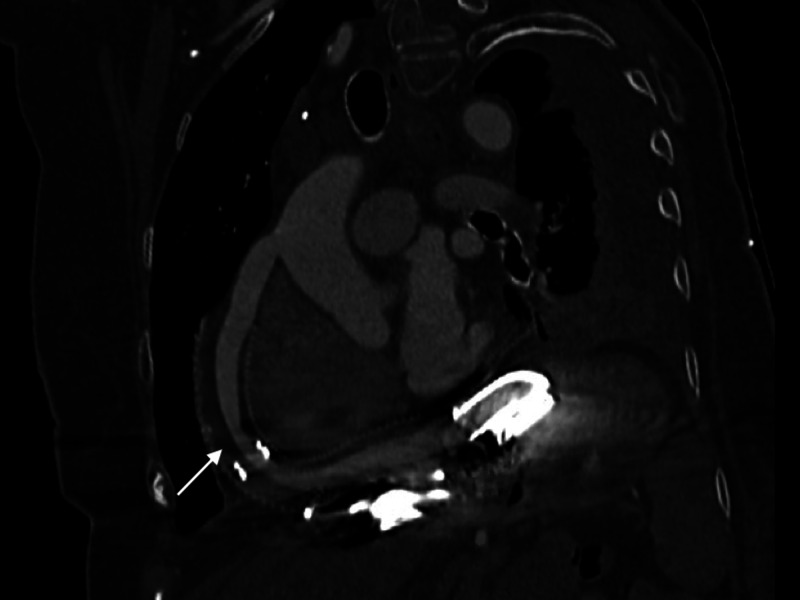
LVAD outflow tract showing narrowing (white arrow) at the midportion consistent with thrombus. LVAD: left ventricular assist device.

Transthoracic echocardiogram was not informative in either case for diagnosis. Computerized tomography of the chest, abdomen, and pelvis during the second admission showed moderate thrombus in the LVAD outflow tract (Figure 3).

Management

For both admissions, she was assessed to be at very high risk for biventricular HVAD exchange or explantation due to her co-morbidities. She had previously decided that she would not want cardiopulmonary resuscitation in the setting of a cardiac arrest and although she was amenable to systemic thrombolytics, she did not want invasive procedures on either occasion. On both admissions, the initial management strategy was through high-intensity heparin with high PTT 70-90 or bivalirudin infusion, which failed to resolve the pump thrombosis based on continued elevated LDH levels and power spikes. After shared decision making, the clinical team proceeded with thrombolysis: 5 mg IV bolus of alteplase followed by a 3 mg/hour infusion for 10 hours given through a central line [6]. This was followed by heparin infusion as a bridge to warfarin with increased INR goals 2.0-3.0 (first admission) and 2.5-3.5 (Admission 2). She was discharged on clopidogrel after the second pump thrombosis.

Follow-up

Three months later she re-presented due to low flow alarms from her RVAD. Her LDH was within the normal range at 212 U/L, and INR therapeutic at 3.4. She had no increase in power output. A mechanical obstruction was suspected given the lack of signs of hemolysis. She was taken to the catheterization lab and an amplazter wire was unable to be passed through the RVAD suggesting thrombosis. The decision was made to decommission the RVAD and the driveline was cut. She was discharged home after RVAD decommissioning and initially did well. Four months later she presented to the hospital with supra-therapeutic INR and intracranial hemorrhage. She transitioned to hospice care and died peacefully shortly thereafter. 

## Discussion

Bilateral HVAD is rarely used as destination therapy [1]. Management of VAD complications without transplant as an option is challenging [7]. Risk factors for developing VAD thrombosis are shown in Table 1 [7,8].

**Table 1:** Risk factors for developing VAD thrombosis. VAD: ventricular assist device.

Patient factors	Pump factors	Management factors
Hypercoagulable state, inherited factors, sepsis, atrial fibrillation, low flow state	Pump heating, inflow cannula malposition, flow dynamics, pump speed, shear stress	Low pump flow, inflow cannula malposition, inadequate anticoagulation

Pump thrombosis incidence for the HVAD device ranges from 0.14 to 0.22 events per patient-year [9]. The presentation varies significantly ranging from asymptomatic to hemodynamic collapse. [10] The diagnosis can be made based on evidence of changes in two out of three INTERMACS criteria: (i) change in HVAD parameters including power spike, or changes in flow and pulsatility; (ii) laboratory evidence of hemolysis with elevated LDH, indirect bilirubin, or plasma free hemoglobin; (iii) heart failure symptoms. Pump thrombosis was diagnosed in our patient based on elevated LDH/plasma free hemoglobin and LVAD power spikes. Both admissions involved pump thrombosis of the LVAD as shown by the measured power and estimated flow parameters of the LVAD and also based on the CT images showing thrombosis in the outflow tract of the LVAD. The etiology was likely pump-related factors as her INR was greater than 2 for both admissions.

A right heart catheterization was not done in our patient as there was high confidence in the diagnosis of pump thrombosis and did not think this would change management. Echocardiographic ramp study to diagnose pump thrombosis has not been validated in biventricular HVAD. The echocardiographic ramp would be challenging since the two VADs are interdependent on each other. As shown in our patient, CTA can show outflow graft kinking and the cannula position [11,12]. Pump thrombosis diagnosis in RVAD is challenging due to the absent cyclical waveform seen in LVAD as well as lack of the validation of echocardiographic ramp in RVAD. 

Medical treatment options include aggressive intravenous anticoagulation with heparin, bivaluridin, or glycoprotein IIb/IIIa inhibitors. Systemic or catheter-directed thrombolysis as therapy for those who fail these initial medical therapies have been reported as successful in case reports or series [4-6]. Previous studies have demonstrated that medical therapy (thrombolysis, heparin, glycoprotein IIb IIIa inhibitors) was associated with low success (48%) and significant risk of hemorrhagic stroke (21%) in HVAD thrombosis [8]. The most definitive therapy for pump thrombosis is the surgical replacement of the VAD; however, the risk of sensitization with repeat surgeries in those awaiting cardiac transplantation is a challenge. In addition, per INTERMACS data, survival at one-year falls with each pump exchange from 80% to 65% to 50% after the third implant [3]. Similarly, patients with LVAD often already have multiple sternotomies or have a prohibitive risk for repeat sternotomy as was the case in our patient. Successful percutaneous intervention with stenting of the stenosed graft has also been reported [10,12]. Due to the high and sometimes prohibitive risk of pump exchange, medical treatment of pump thrombosis is oftentimes a more favorable conservative approach.

In our patient, we proceeded with systemic thrombolysis because surgical pump exchange was considered prohibitively risky. In theory, catheter-directed lysis might mitigate the risk of bleeding complications associated with systemic thrombolysis; however, catheter delivery was not consistent with her goal of avoiding invasive procedures. Our experiences add to the current literature demonstrating the success of medical treatment of pump thrombosis and provide a rare example of the safety of such treatment in a patient with biventricular support devices.

## Conclusions

In patients who are unable to undergo surgical pump exchange for HVAD pump thrombosis due to prohibitive risk, there is no standard treatment. While the etiology and clinical presentation of pump thrombosis can vary, this well-described complication requires intervention. As summarized above, possible treatments can include intensified anticoagulation, systemic thrombolysis, catheter-directed lysis, percutaneous intervention, and pump exchange.

We have presented a case of a patient with bilateral HVADs as destination therapy, which by itself is an uncommon treatment pathway, who developed signs of recurrent pump thrombosis. She was successfully treated on two separate occasions with systemic thrombolytics. This case demonstrates that systemic thrombolysis can be considered in acute pump thrombosis in bilateral HVAD patients.
